# A group-based lifestyle intervention for diabetes prevention in low- and middle-income country: implementation evaluation of the Kerala Diabetes Prevention Program

**DOI:** 10.1186/s13012-018-0791-0

**Published:** 2018-07-18

**Authors:** Zahra Aziz, Elezebeth Mathews, Pilvikki Absetz, Thirunavukkarasu Sathish, John Oldroyd, Sajitha Balachandran, Suman S. Shetty, K. R. Thankappan, Brian Oldenburg

**Affiliations:** 1 Melbourne School of Population and Global Health, The University of Melbourne, Melbourne, Australia; 2 Department of Epidemiology and Preventive Medicine, Monash University, Melbourne, Australia; 3 WHO Collaborating Centre on Implementation Research for Prevention & Control of NCDs, Melbourne, Australia; 4 Department of Public Health and Community Medicine, Central University of Kerala, Kasaragod, India; 5 School of Health Sciences, University of Tampere, Tampere, Finland; 6 Collaborative Care Systems Finland, Helsinki, Finland; 7 Centre for Population Health Sciences, Lee Kong Chian School of Medicine, Nanyang Technological University, Singapore, Singapore; 8 Achutha Menon Centre for Health Science Studies, Sree Chitra Tirunal Institute for Medical Sciences and Technology, Thiruvananthapuram, India

**Keywords:** Implementation evaluation, Type 2 diabetes mellitus, Diabetes prevention, Lifestyle interventions, Behavioural interventions, Low- and middle-income countries, Peer support, RE-AIM framework, PIPE impact metric

## Abstract

**Background:**

While several efficacy trials have demonstrated diabetes risk reduction through targeting key lifestyle behaviours, there is a significant evidence gap in relation to the successful implementation of such interventions in low- and middle-income countries (LMICs). This paper evaluates the implementation of a cluster randomised controlled trial of a group-based lifestyle intervention among individuals at high-risk of developing type 2 diabetes mellitus (T2DM) in the state of Kerala, India. Our aim is to uncover provider-, participant- and community-level factors salient to successful implementation and transferable to other LMICs.

**Methods:**

The 12-month intervention program consisted of (1) a group-based peer-support program consisting of 15 sessions over a period of 12 months for high-risk individuals, (2) peer leader (PL) training and ongoing support for intervention delivery, (3) diabetes education resource materials and (4) strategies to stimulate broader community engagement. The evaluation was informed by the RE-AIM and PIPE frameworks.

**Results:**

Provider-level factors: Twenty-nine (29/30, 97%) intervention groups organised all 15 sessions. A 2-day PL training was attended by 51(85%) of 60 PLs. The PL handbook was found to be ‘very useful’ by 78% of PLs. Participant-level factors: Of 1327 eligible individuals, 1007(76%) participants were enrolled. On average, participants attended eight sessions. Sixty-eight percent rated their interest in group sessions as ‘very interested’, and 55% found the group sessions ‘very useful’ in making lifestyle changes. Inconvenient time (43%) and location (21%) were found to be important barriers for participants who did not attend any sessions. Community-level factors: Community-based activities reached to 41% of the participants for walking groups, 40% for kitchen garden training, and 31% for yoga training. PLs were readily available for support outside the sessions, as 75% of participants reported extracurricular contacts with their PLs. The commitment from the local partner institute and political leaders facilitated the high uptake of the program.

**Conclusion:**

A comprehensive evaluation of program implementation from the provider-, participant- and community-level perspectives demonstrates that the K-DPP program was feasible and acceptable in changing lifestyle behaviours in high-risk individuals. The findings from this evaluation will guide the future delivery of structured lifestyle modification diabetes programs in LMICs.

**Trial registration:**

Trial registration: Australia and New Zealand Clinical Trials Registry ACTRN12611000262909. Registered 10 March 2011.

## Background

More than 415 million people currently have type 2 diabetes mellitus (T2DM) worldwide. This number is expected to increase such that by 2040, half a billion people (642 million cases) between the ages 20 and 79 years worldwide will be affected [1–3]. About 75–80% of people with T2DM live in low- and middle-income countries (LMICs) [3–5]. Globally, India has the second largest number of people with T2DM (> 69 million) after China, and this is predicted to double by 2040 [5–7]. India also has the largest number of individuals (36.5 million) with impaired glucose tolerance (IGT) and prediabetes [5], conditions with a high risk of progression to T2DM [8]. Notably, many of these individuals with IGT and prediabetes are unaware of their condition and therefore are at high risk of developing diabetes complications [5]. The high burden of T2DM puts an enormous burden on affected individuals, their families, and healthcare systems in LMICs [9]. This demands urgent action from program planners and policymakers to prevent and control T2DM. [6] Furthermore, while the management of those already diagnosed with T2DM is important, delaying the onset of the disease in high-risk individuals is urgently needed [10], particularly in LMICs.

A number of large randomised controlled trials (RCTs) from the USA [11], China [12], Finland [13], India [14] and Japan [15] have now demonstrated that lifestyle interventions can prevent T2DM by up to 60% among individuals with IGT. Furthermore, these effects have been maintained for up to 20 years [16]. Among the diabetes prevention trials in high-risk populations conducted to date, few have been undertaken in LMICs [14, 17–19]. For the successful translation of effective programs, particularly in resource-constrained settings, implementation and dissemination are needed to inform practice as well as policy [20–22]. Implementation research focuses on the generation of evidence concerning the processes affecting program implementation in different settings and contexts and future program scalability. The findings from such evaluations are also beneficial when assessing the transferability and scalability of the intervention to other settings. Implementation evaluation can also help identify ‘why’ and ‘how’ interventions work in real-world settings [23]. Indeed, understanding the enablers of and barriers to program adoption can inform understanding of program transferability to other settings and scalability to other populations [21, 24, 25]. Hence, a comprehensive understanding of implementation evaluation can inform the future transferability, scalability, and dissemination of effective programs [26, 27].

The Kerala Diabetes Prevention Program (K-DPP) was a group-based peer-support lifestyle intervention aimed at reducing the risk of T2DM in high-risk individuals. The primary outcome was the incidence of T2DM at 24 months. Secondary aims included changes in clinical, biochemical and behavioural risk factors known to increase diabetes risk, including weight, waist circumference, waist-to-hip ratio, systolic and diastolic blood pressure, body composition measures, plasma glucose, HbA1c, total cholesterol, LDL cholesterol, tobacco use, alcohol use, diet and physical activity at 24 months [28]. The intervention program involved four core components: (1) a group-based peer-support program consisting of 15 sessions for high-risk individuals, (2) peer-leader training and ongoing support for intervention delivery, (3) diabetes education resource materials and (4) strategies to stimulate broader community engagement [7].

The primary effectiveness outcomes are reported elsewhere [28]. At 24 months, the incidence of T2DM was 14.9% in the intervention arm as compared to 17.1% in the control arm (*p* = 0.36). This paper reports the findings of the implementation evaluation on provider-, participant- and community-level factors, guided by the Glasgow’s RE-AIM framework [29] and Pronk’s PIPE Impact Metric [30].

This implementation evaluation aims to understand more about the process of delivering a structured lifestyle management program in a resource-constrained setting such as India and how to improve the future delivery of such programs in LMICs.

## Methods

### Study design, setting and recruitment

The study protocol, baseline characteristics of participants and main study outcomes have been published [7, 28, 31]. Briefly, the study was a cluster RCT, implemented in 60 polling areas (electoral divisions) of Neyyattinkara taluk (sub-district) in Trivandrum district of Kerala state, India. Neyyattinkara taluk has four legislative assembly constituencies (LACs). Sixty polling areas (15 from each LAC) were randomly selected, and 30 polling areas were randomly allocated to the intervention and control arms in a 1:1 ratio. A total of 5517 individuals between the ages of 30 and 60 years (approximately 92 individuals in each of the 60 polling areas) were randomly selected and approached through home visits. Initially, we aimed to select 80 individuals in each polling area. However, this number varied from 42 to 212 individuals depending on the availability of records in the electoral roll, incorrect addresses, emigration, deaths or unavailability of participants at the house at the time of contact. From our pilot study findings published elsewhere [7, 32], we found that participation by men was lower than women selected for the pilot program. Hence, we screened more men (64%) than women (36%) to achieve a gender balance in our study.

After obtaining written informed consent, a screening questionnaire consisting of eligibility criteria and the Indian Diabetes Risk Score (IDRS) was administered [7, 33]. Eligible participants comprised individuals aged 30–60 years, who were able to speak, read and write Malayalam (the local language). Participants were excluded if they had a prior diagnosis of T2DM, had other chronic disease(s) that would impede the participation in the trial, were currently using medications known to affect glucose tolerance or were pregnant. Participants who met the eligibility criteria with an IDRS value of ≥ 60 were invited to attend a mobile clinic in their local community for an oral glucose tolerance test (OGTT) and further assessment. Those at high risk of developing T2DM based on an IDRS value ≥ 60, and without diabetes on OGTT, were invited to participate in the study. Those diagnosed with diabetes were referred to health care facilities for further management. Participants completed assessments at baseline, 12 and 24 months. The control arm participants received an education booklet with information about diabetes and its risk factors and advice on standard lifestyle modifications. The intervention arm participants received a 12-month intensive lifestyle intervention program.

### Intervention program

The details of the K-DPP intervention program have been published previously [7, 31, 32]. Briefly, the program objectives included increasing the consumption of fruit, vegetables and fibre; reducing the intake of carbohydrates with high glycaemic index and total and saturated fats; increasing physical activity; reducing tobacco use; reducing alcohol consumption; and setting realistic goals for weight loss and other lifestyle risks. The K-DPP intervention program consisted of the following four core components: (1) Group-based peer-support program consisting of 15 sessions for high-risk individuals: The intervention participants received a 12-month intervention program consisting of 15 sessions, aimed at targeting and monitoring lifestyle behaviours. The first session was an introductory group session (lasted for 60–90 min). Two half-day diabetes prevention education sessions (DPES) were delivered by experts in the field of diabetes, nutrition and physical activity. Twelve group sessions (~ 60–90 min each) were held ranging from 10 to 23 participants (median 17) at local venues such as community centres, local reading rooms, community schools and peer leader’s homes. Table 1 describes the content of each of the 15 sessions.

**Table 1:** K-DPP group-session content

Sessions	Facilitate by	Content
Session 1—Introductory session	K-DPP team	• Program description • Participant handbook distribution • Peer leader selection
Session 2—Diabetes Prevention Education Session (DPES1)	Expert panel (specialist advisors on diabetes)	• Information on type 2 diabetes mellitus (T2DM) • T2DM risk factors • T2DM management
Session 3	Peer leaders	• Setting ground rules • Setting key targets • Self-assessment
Session 4—(DPES2)	Expert panel	• Modifying the risk factors to prevent T2DM
Session 5	Peer leaders	• Goal setting for diet and physical activity
Session 6	Peer leaders	• Goal setting for tobacco and alcohol
Session 7–11	Peer leaders	• Ongoing goal monitoring • Content review
Session 12	Peer leaders	• Interim evaluation of participants’ benefits
Session 13–14	Peer leaders	• Ongoing goal monitoring • Content review
Session 15	Peer leaders	• Overall program evaluation • Ongoing support

The peer support component was adapted from the US Peers-for-Progress program [34, 35]. (2) Peer-leader training and ongoing support for intervention delivery: During the inaugural session, each group identified and nominated peer leaders among themselves, based on their social credibility, willingness to lead the group and their acceptability to other group members. The K-DPP team delivered a 2-day training session for peer leaders before session 3 which took place after the first DPES given by experts (see Table 1). The peer leaders also received a 2-day refresher training after session 8. In order to support peer leaders for intervention delivery, a local resource person (LRP) was nominated for each group. LRPs are community mobilisers mostly Accredited Social Health Activists (ASHAs) who were nominated by local self-government bodies, called the Panchayats, to support implementation. The responsibilities of LRPs included assisting peer leaders in organising the group sessions and community-based activities, following up with group participants and encouraging them to attend the group sessions and advocating for the program among local community-based organisations. The LRPs also attended peer-led sessions as an observer and supported the peer leaders, whenever possible. The intervention team provided ongoing support to peer leaders throughout the intervention period. The team had regular telephone contact with each peer leader before and after each group session in order to assist with preparation prior to or with reflection and review following each session. In addition, two face-to-face meetings were organised to facilitate knowledge exchange and sharing of learnings among peer leaders. (3) Diabetes education resource materials for participants and peer leaders: Each participant received a Participant Handbook containing information on diabetes risk factors and its prevention. Each participant also received a Participant Workbook to guide them through group sessions with self-monitoring of lifestyle behaviours, goal setting and review and ongoing group support. The participants were also given a non-elastic measuring tape and taught to measure their waist circumference to assess the progress towards their weight reduction. Peer leaders were provided with a Peer-leader Handbook which outlined the group sessions’ objectives, along with activity guide and exercises to prepare them for conducting the sessions. In addition to measuring tapes, the peer leaders were also given measuring cups and spoons to assist them in educating the participants about the correct serving sizes for foods such as rice, oil, sugar and salt. (4) Strategies to stimulate broader community engagement: The group-based sessions were complemented by a range of community engagement strategies to reinforce the importance of adopting and maintaining healthy lifestyle behaviours learnt in the group sessions in the community. The community-based activities were organised by peer leaders with the support of LRPs outside the peer-group sessions. As part of this strategy, individuals were encouraged to participate in various activities in the local neighbourhoods such as walking groups, kitchen garden training and yoga clubs. The participants were encouraged to bring family members and other community members to take part in these activities.

### Ethics approval

The study was approved by the Institutional Ethics Committee of the Sree Chitra Tirunal Institute for Medical Sciences and Technology, Trivandrum, Kerala, and by the Human Research Ethics Committees of Monash University, Australia, and the University of Melbourne, Australia. The study was also approved by the Health Ministry Screening Committee of the Government of India.

### Design of the implementation evaluation

#### Evaluation frameworks

The implementation evaluation was guided by the Glasgow’s RE-AIM framework [29] and Pronk’s PIPE Impact Metric [30]. RE-AIM includes five dimensions, i.e. reach (R), effectiveness (E), adoption (A), implementation (I), maintenance (M). The PIPE Impact Metric has four evaluation components, i.e. penetration (P), implementation (I), participation (P), effectiveness (E) [30]. Both the RE-AIM and PIPE employ provider-level as well as participant-level factors. In PIPE, these user levels are separate (Penetration and Implementation for provider, and Participation and Effectiveness for participant). In RE-AIM, some dimensions (i.e. ‘adoption’ and ‘maintenance’) include both user levels, which makes it difficult to identify exactly which program element would need to be addressed to improve the program. Furthermore, several reviews have shown that these two RE-AIM dimensions are largely underreported [36–42] and that their validity is often uncertain due to poor or varying operationalisation of the different dimensions. For example, RE-AIM defines ‘participant-level maintenance’ as ‘the effect of the intervention on the outcome at 12 or more months’ [29], which overlaps with the definition of ‘effectiveness’ component.

Similarly, ‘provider-level maintenance’ is ‘the extent to which the intervention became part of routine organisational practice’ [29]. This dimension is important in assessing the sustainability of the program. However, traditionally, it is not measured as part of the implementation process, and it usually takes considerable time to adopt a new research program in routine practice. This is particularly the case in resource-constrained settings; hence, this dimension of RE-AIM often remains unreported. Also, some reviews found that the definitions of the ‘reach’ and ‘adoption’ components were overlapping [43].

In undertaking this evaluation, we incorporated elements of both the RE-AIM and PIPE frameworks. When combined, the two models complement each other by enhancing the understanding of the context in which the intervention was implemented. However, in order to identify potential enablers and barriers to implementation and future scalability, evaluating the community-level factors is vital. Hence, for the purpose of this evaluation, some components from both the frameworks were adapted in the context of K-DPP. We have then included additional components such as community-level factors and barriers to participation and intervention delivery.

**Fig. 1:**
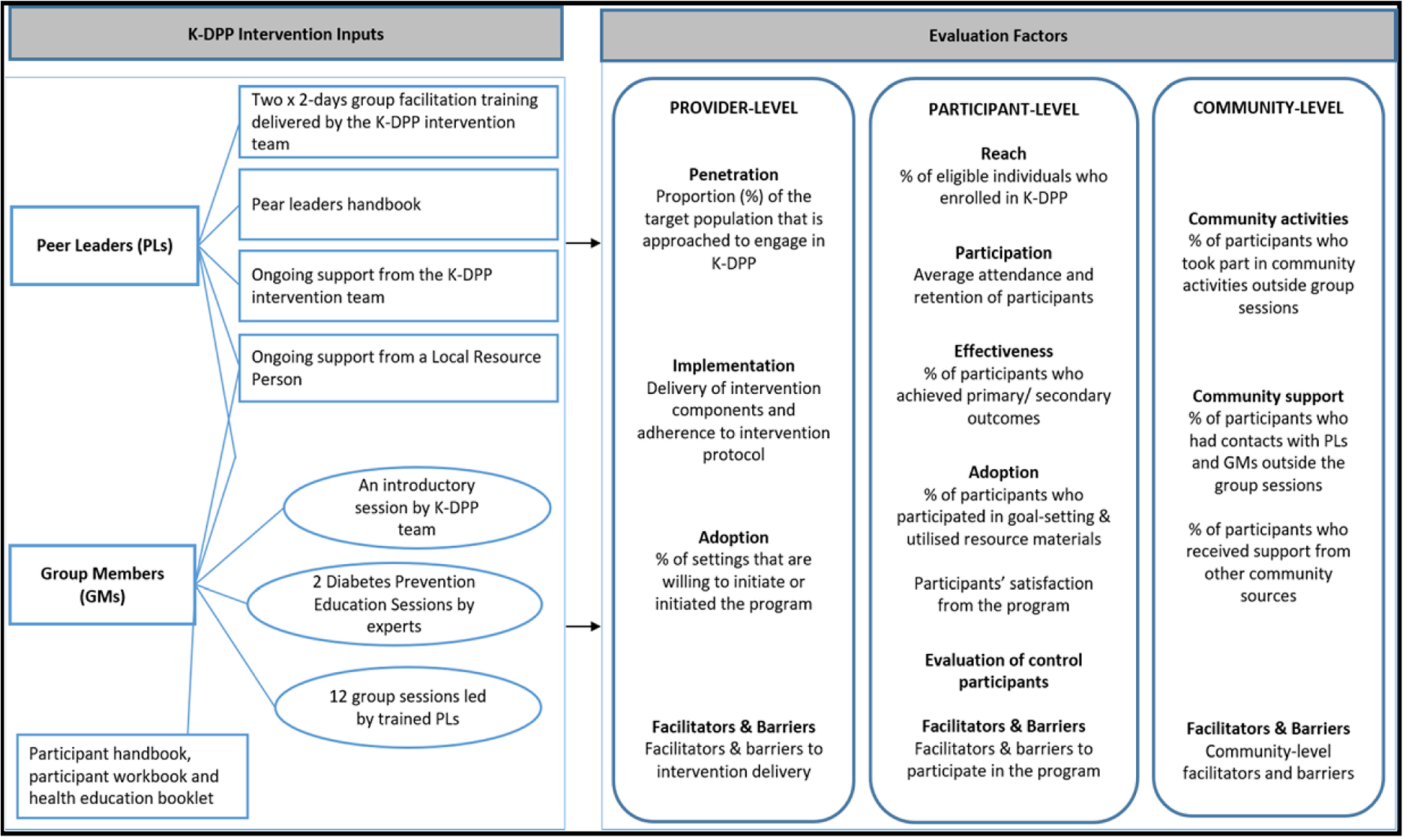
K-DPP intervention inputs and implementation evaluation factors

This paper assesses *provider-level factors* (penetration into target population, implementation, setting-level adoption, facilitation and barriers to intervention delivery), *participant-level factors* (program’s reach, participation, individual-level adoption, participants’ satisfaction, facilitators and barriers to participation) and *community-level factors* (community activities, community support and community-level facilitators and barriers). Figure 1 shows the K-DPP intervention inputs and evaluation measures.

### Data collection and analysis

Table 2 describes the evaluation measures used for assessing the provider-, participant- and community-level factors; lists data sources including data collection tools specifically designed for the K-DPP intervention; and indicates the timing of the data collection.

**Table 2:** K-DPP implementation evaluation measures, data sources and their calculations

Evaluation measures	Description	Data sources	Collected by	Time points	Data analysis
Provider-level factors					
Penetration	Penetration into the target population	Recruitment records	Intervention team	At the beginning of the program	Numerator: # of individuals approached or invited to engage in the program Denominator: # of individuals in the target population
Implementation	Delivery of intervention components and adherence to intervention protocol	Peer-leader training evaluation questionnaire, attendance sheet and group session reports completed by peer leaders	Peer leaders; and/or intervention team (for attendance)	Training evaluation at the end of the training; Attendance record during each session; and Group session reports at the end of each session	Assessment of intervention components (peer leader training, group sessions, resource material and ongoing support) to ascertain their delivery as planned
Adoption	Provider-level (setting-level) participation	Group session reports completed by peer leaders	Peer leaders	At the end of each session	Numerator: # of polling areas enrolled in the program Denominator: total number of polling areas randomly selected for inclusion in the program, at the beginning of the program
Facilitators and barriers	Provider-level facilitators and barriers	Group session reports completed by peer leaders	Peer leaders	At the end of each session	Assessment of facilitators and barriers to intervention delivery
Participant-level factors					
Reach	Proportion of eligible individuals enrolled in the program	Recruitment records	Intervention team	At the beginning of the program	Numerator: # of participants enrolled in the program Denominator: # of eligible individuals
Participation	Participants’ attendance in the program	Attendance sheet/recruitment records	Peer leaders and/or intervention team	During each session	Average attendance in 15 group-based sessions
	Participants’ retention in the program			As needed	Numerator: # of participants who provided measurements at 12-months Denominator: # of participants enrolled in the program
Effectiveness	Effectiveness according to primary and secondary outcomes	Clinical measurements records	Intervention team	At baseline, 12 months and 24 months	Numerator: # of participants who developed diabetes at 24 months Denominator: # of individuals who provided measurements at 24 months Changes in clinical and behavioural characteristics from baseline to 12 and/or 24 months.
Adoption	Participation in goal setting for behaviour change	Intervention participants evaluation questionnaire	Intervention participants	At 12 months	Numerator: # of participants who made change(s) to their lifestyle to meet their goals Denominator: # of participants who set goals for behaviour change Numerator: # of participants who were willing to adhere to the lifestyle change after the intervention Denominator: # of participants who made lifestyle change(s)
	Utilisation of diabetes education resource materials				Numerator: # of participants who utilised the diabetes education resource materials Denominator: # of participants who received the diabetes education resource materials
	Participants’ satisfaction with the program				Participants’ satisfaction with the peer-led groups based on a series of questions asked at the end of 12-months
Evaluation of control participants	Evaluation of control participants at 12-months	Control participants evaluation questionnaire	Control participants	At 12 months	Participant’s use of health information booklet and behaviour changes made by participants over the duration of the intervention
Facilitators and barriers	Participant-level facilitators and barriers	Intervention participants evaluation questionnaire	Intervention participants	At 12 months	Assessment of facilitators and barriers to participate in the program
Community-level factors					
Community activities	Participation in community activities outside group sessions	Intervention participants evaluation questionnaire	Intervention participants	At 12 months	Numerator: # of participants who took part in community activities Denominator: # of participants enrolled in the program
Community support	Frequency of additional contacts between peer leader and group member and among group members outside the group sessions	Intervention participants evaluation questionnaire	Intervention participants	At 12 months	Numerator: # of participants who contacted their peer leaders/group members outside the group sessions Denominator: # of participants enrolled in the program An average number of contacts made during the intervention.
	Support received during the intervention period from sources other than K-DPP				Numerator: # of participants who received support from other community sources Denominator: # of participants enrolled in the program
Facilitators and barriers	Community-level facilitators and barriers to participation and/or intervention delivery	Ongoing interactions between peer leaders and the K-DPP intervention team including telephone calls and face-to-face meetings Group session reports completed by peer leaders	Intervention team	Ongoing	Assessment of community-level facilitators and barriers

All quantitative data were analysed using IBM Corp. Released 2017. IBM SPSS Statistics for Windows, Version 25.0. Armonk, NY: IBM Corp. All non-numerical data was coded and categorised into similar themes in SPSS and were then analysed using descriptive statistics.

## Results

This section presents the results based on the evaluation measures described in Table 2, for the provider-, participant- and community-level factors.

### Provider-level factors

#### Penetration

Of the 5517 individuals identified from the electoral roll of selected polling areas, 3689 (67%) individuals were contacted during home visits. More women (69%) were contactable than men (66%). The remaining individuals could not be contacted due to reasons such as incorrect addresses, immigration, deaths or unavailability of participants at the house at the time of contact.

#### Implementation

The findings of the intervention program delivery based on the four core components are given below.

1. A group-based peer-support program for high-risk individuals:
  *Groups and sessions delivered*: Twenty-nine [29] out of 30 intervention groups organised all 15 group-based sessions over the 12 months duration. One group did not participate in an intervention program due to lack of support from the local leadership in organising sessions in the locality. Hence, the group did not continue. The individuals from this group who were interested in participating in the program were then combined with the other neighbouring groups.
  *Facilitation style as per protocol*: To assess the autonomy supportive behaviours of peer leaders, we asked the group members a set of questions on the perceptions of autonomy support. Reflecting on the group sessions, 78% stated that they were always free to choose the kind of lifestyle changes they wanted to make (and were not being enforced by peer leaders). Seventy-two percent and 21% of the participants stated that their opinions were understood and appreciated in the group from ‘always’ to ‘most of the times’, respectively. More than 90% of participants stated that their peer leaders encouraged them to ask questions in group sessions, understood the way they see their lifestyle and acknowledged the way they wanted to make changes to their lifestyle, without being judgmental.
2. Peer-leader training and ongoing support for intervention delivery:

Of the 60 peer leaders (two per group; one male and one female) who were originally identified for training, nine leaders did not attend training due to reasons including emigration for job, caregiver responsibility at home and lack of time due to other commitments. A 2-day peer-leader training was attended by the remaining 51 peer leaders (85%). At the end of the training, 80% of those in training completed the evaluation questionnaire. Sixty-three percent and 34% of peer leaders stated that the training had prepared them for leading the groups from ‘very well’ to ‘somewhat’, respectively. A 2-day refresher training was also organised by the intervention team. The training was attended by 48 (94% of 51) peer leaders. The LRPs and the K-DPP intervention team provided ongoing support to all peer leaders every month including telephone contact before and after each group session, face-to-face meetings and assistance in organising the sessions. LRPs also assisted peer leaders in organising community-based activities.

3. Diabetes education resource materials for participants and peer leaders:

All participants were provided with a Participant Handbook written in the local language (Malayalam). All participants were also given Participant Workbook, which was regularly used during group sessions. The participants also received a non-elastic measuring tape and were taught to measure their waist circumference to assess the progress towards their weight loss goals. All peer leaders used their handbook while preparing for group sessions. Seventy-eight percent (78%) found it ‘very useful’, and 22% found it ‘somewhat useful’. Seventy-seven percent and 23% gathered ‘a lot of information’ and ‘some information’ from the Peer-leader Handbook. Peer leaders were also asked their views on the usefulness of Participant Handbook. Fifty-six percent and 44% peer leaders found it ‘very useful’ and ‘somewhat useful’, respectively.

4. Strategies to stimulate broader community engagement:

To assist participants in attaining behaviour change goals, peer leaders with the support of LRPs organised various community-based activities outside the peer-group sessions, such as yoga sessions, walking groups, and kitchen garden training. The participants were encouraged to bring family members and other community members to take part in these activities. A number of local community organisations (such as resident’s associations, arts and sports club and religious groups) also collaborated in promoting these activities. The K-DPP team also collaborated with the State Agriculture Department for facilitating the kitchen garden training. Furthermore, the local communities organised various activities to stimulate community’s interest and awareness towards healthy living. Some of these activities included diabetes quiz competition, healthy living drawing competition, essay writing on diabetes, cooking competitions and sporting activities for children. Some of the community groups also conducted seminars delivered by doctors from the local primary healthcare clinics. The data on some of the community engagement activities is provided in the ‘Community-level factors’ section below.

#### Adoption (provider-level)

Provider-level adoption was calculated based on the number of polling areas that were enrolled in the program. Of 60 randomly selected, a total of 59 (98%) polling areas participated as either intervention [29] or control [30] arms in the K-DPP intervention.

#### Facilitators and barriers to intervention delivery

A majority of peer leaders (73%) expressed that the participants were ‘very motivated’ during the sessions, which created a conducive environment for learning. Peer leaders were asked to share their experience about the most positive aspects of the intervention delivery. Adoption of a healthy diet and physical activity by participants, participants’ motivation and interest in group sessions and diabetes education through DPES and monthly group sessions were the most positive aspects of the intervention.

The inclusion of more group activities such as arranging regular physical activity classes for group participants was seen by peer leaders as one of the main areas for further improvement. Some peer leaders stated that their groups needed more sessions on diabetes prevention by specialists and experts, whereas some stated that they wanted to see more involvement of the intervention team in delivering the sessions. A small proportion of peer leaders (6%) stated that they had challenges with facilitating group sessions. These challenges included not being able to start group sessions on time due to participants not arriving on time, some participants not being fully engaged in the session and the timing of some group sessions which was not always convenient for all participants.

### Participant-level factors

#### Reach

The detailed consort diagram and participants’ demographics have been published previously [31]. Briefly, 60 polling areas were selected and randomised into control [30] and intervention arms [30]. Of the 3421 individuals assessed for eligibility, 835 (24%) did not satisfy the eligibility criteria. The remaining 2586 (76%) were screened with the IDRS tool. Of these, 1057 (41%) were excluded due to having IDRS score < 60. The remaining 1529 (59%) had an IDRS score > 60 and were invited for further testing using OGTT. Of these, 320 (21%) declined an OGTT test. Of the remaining 1209 who completed OGTT, 202 (17%) were diagnosed with T2DM. The remaining 1007 individuals (53% men) were enrolled in the trial (500 in the intervention arm and 507 in the control arm). Hence, out of a total of 1327 eligible individuals, 1007 (76%) were enrolled in the intervention or control arms.

#### Participation

On an average, the participants attended eight sessions (median 9, mode 14). Almost half (49%) of the participants attended 10 or more sessions with 11% attending all 15 sessions. Ten percent of the participants did not attend any sessions. At the end of 12 months, the retention rate was 97 and 98% in the intervention and control arm, respectively.

#### Effectiveness

##### Primary and secondary outcomes

The detailed outcomes are reported elsewhere [28]. Briefly, at 24 months, the incidence of T2DM was 14.9% in the intervention arm as compared to 17.1% in the control arm. The relative risk was 0.88 (95% confidence interval 0.66 to 1.16), *p* = 0.36. At 24 months, the reduction in IDRS score was 1.50 points higher in the intervention arm as compared to the control arm (*p* = 0.022 for difference). The intervention participants were 83% more likely to consume ≥ 5 servings of fruit and vegetables per day (*p* = 0.008) and 23% less likely to consume alcohol compared with the control participants (*p* = 0.018) at 24 months. Also, the amount of alcohol consumed was significantly lower among the intervention participants (*p* = 0.030). No adverse events related to the intervention were noted [28].

#### Adoption (participant-level)

The data was collected on goal setting and tracking to assess the extent of the strategies learnt and adopted during the intervention and the likelihood of adherence after the intervention. The participants were encouraged to set goals if they had risk factors and were willing to make lifestyle changes to reduce their risk of developing T2DM. Not all participants had all risk factors. For example, not all participants were using tobacco and/or alcohol. Hence, the goal setting for each lifestyle behaviour was only applicable to a subset of the participants. Table 3 shows the data for goal setting, self-reported behaviour change over the duration of the intervention and the likelihood of adherence to the change in future.

**Table 3:** Goal setting, self-reported behaviour change and likelihood of adherence to the change in future*—*intervention participants

Goal	I have set this goal (*N*)	Since joining K-DPP, I have made lifestyle changes to achieve this goal (%)	I will continue to make these changes after the intervention		
			Very likely (%)	Likely (%)	Not sure (%)
Improving diet	346	99	83	15	2
Increasing physical activity	306	96	67	32	1
Reducing smoking/tobacco	51	76	82	18	–
Reducing alcohol consumption	62	98	56	34	10

Of 346 participants who set a goal for improving diet, almost all (99%) indicated that they had made lifestyle changes during the intervention to achieve this goal. When asked about the specific changes that they had made, a majority of participants stated that they had reduced the consumption of oil and fatty food (72%), increased fruit and vegetable intake (60%), reduced rice consumption (57%) and/or made other dietary changes (54%) such as replacing white rice with wheat-based choices or decreasing the frequency of meat consumption. About 44% of participants stated that they had reduced their sugar intake as part of adopting a more healthy lifestyle.

Similarly, 96, 76 and 98% of those who set goals for increasing physical activity, reducing smoking/tobacco and reducing alcohol consumption, respectively, stated that they had made lifestyle changes to achieve their goals. More than 90% of participants indicated they were willing (‘very likely’ or ‘likely’) to continue to making these changes following the completion of the structured intervention at 12 months. Reducing smoking/tobacco was the least adopted behaviour change among those who set goals for it.

Ninety percent of the participants reported using the Participant Handbook during the program from ‘very often’ (12%), ‘often’ (26%) and ‘sometimes’ (52%), whereas 10% of participants did not use their handbooks. Of those who used the Handbook, 52, 40 and 9% found it to be ‘very useful’, ‘useful’ and ‘somewhat useful’, respectively. About 61% of the participants used measuring tape to measure their waist circumference, whereas 39% did not use the measuring tapes.

#### Intervention participant’s satisfaction from group sessions

Overall, 55, 32 and 4% of participants stated that the monthly group sessions have been ‘very useful’, ‘useful’ and ‘somewhat useful’, respectively, whereas 8% felt that the sessions were not useful to them. Almost all participants (98%) reported that they had shared their learnings through group sessions with their family members.

#### Evaluation of control participants at 12 months

A total of 495 (98%) control participants completed the evaluation questionnaire at 12 months. Eighty-three percent of the control participants stated that they had seen the health information booklet, and of these, 92% stated that they had used, read or looked at the booklet in the last 12 months ‘very often’ (3%), ‘often’ (27%) and ‘sometimes’ (62%). Control participants were asked whether they had made any changes in relation to changing their diet, increasing their physical activity, reducing smoking/tobacco use or reducing alcohol intake. Table 4 shows the changes made by the control participants and the likelihood of adherence to the change in future.

**Table 4:** Self-reported behaviour change and likelihood of adherence to the change in future*—*control participants

Goal	I have made changes (*N*)	I will continue to make these changes in future		
		Very likely (%)	Likely (%)	Not likely/not sure (%)
Improving diet	400	95.5	4	0.5
Increasing physical activity	322	97	3	–
Reducing smoking/tobacco	57	95	2	3
Reducing alcohol consumption	75	81	11	9

#### Facilitators and barriers to participation

Among those participants who enrolled in the program and attended one or more group sessions, a majority stated that the location (85%) and timings (77%) of the group sessions were either ‘very convenient’ or ‘convenient’. Fifteen percent and 23% found the location and time of the group session either ‘somewhat convenient’ or ‘not convenient’. Participants rated their interest in group sessions from ‘very interested’ (68%) to ‘interested’ (28%). Only 4% of the participants stated that they were either ‘somewhat interested’ or ‘not interested’, and 6% stated that they did not look forward to meeting their peer leaders or other group members in the group sessions. The participants were asked whether there was anything they did not like about the sessions. One participant felt that there was no unity or agreement on common themes, among their group members.

We also assessed the 12-month evaluation data from 48 participants (10% of total enrolled) who were enrolled in the intervention but did not attend any sessions. Of these 48 participants (71% male), 44 and 21% stated that the time and location of the group sessions were not convenient for them, respectively. Fifteen percent stated that they were not interested in attending group sessions, whereas 40% stated that the sessions were not useful for them.

### Community-level factors

#### Other community activities

The participants were asked whether they had participated in any community-based activities outside the group session over the duration of the intervention. Forty-one, 40 and 31% of individuals participated in the walking groups, kitchen garden training and yoga training, respectively.

#### Community support

Seventy-five percent of the participants had contacts with their peer leaders outside the group sessions, over the duration of the intervention. On an average, 11 contacts were recorded between peer leaders and participants. The nature of these contacts included touching base on the missed sessions or seeking further clarification on topics that were discussed during the group meetings. Similarly, 70% of the participants had contacts (mean = 9 contacts) with other group members outside the group sessions. During these contacts, participants discussed the content of the session and encouraged each other to attend future sessions.

Participants were asked whether they have received any support from other community sources in making lifestyle changes over the duration of the intervention. Forty-nine and 31% of the participants stated that they have received ‘a lot of support’ from their family members and friends, respectively.

#### Community-level facilitators and barriers

In some intervention communities, the commitment of the local political leaders emerged as one of the major facilitators for high uptake of the program.

A few peer leaders stated that while they regularly discussed the importance of physical activity, as such, there were no suitable public places for conducting these activities as a group. Another barrier was reported from one intervention group that underwent the baseline screening and assessment but did not participate in an intervention program due to lack of support by the local community. Hence, the group did not continue.

## Discussion

As far as we are aware, K-DPP is one of the first group-based, peer-support lifestyle intervention programs for diabetes prevention in a LMIC. This paper describes the detailed evaluation of this program for its feasibility, reach, adoption and implementation. In the following discussion, we summarise and discuss the key findings of the evaluation and identify the key strengths and shortcomings of this implementation evaluation. Furthermore, we discuss the implications of our findings for diabetes prevention program planning and implementation in other similar LMIC settings.

### Summary of key findings

Table 5 summarises the key success factors of the K-DPP intervention and the implementation components that need to be improved.

**Table 5:** Key findings of the K-DPP implementation evaluation

Key success factors	Need to improve
• Home visits that guaranteed reasonably high reach, made by a trustworthy community-based organisation; • Peer leader training program that was feasible to deliver, easy enough to receive and relatively short but managed to provide skills needed, as perceived by peer leaders and participants; • Educational resource materials were perceived useful and actively used by peer leaders and participants; • Support provided by family and friends; • Involvement of experts to provide information that targeted knowledge gaps that peer leaders and participants also found salient; • K-DPP intervention team’s ongoing support to peer leaders; • Local resource person with a broad role to support peer leaders in practical arrangements as well as link with community; and • Engagement of community organisations and members to practical activities.	• Timing and venue for peer leaders training to increase accessibility; • Timing and location for group sessions, possibility to replace/complement face-to-face meeting with other delivery modes; • Inclusion of additional group activities such as arranging regular physical activity classes for group participants; and • Inclusion of additional sessions from diabetes experts.

The evaluation of the *provider-level factors* shows that the implementation was delivered as planned. The peer-leader training was well received and equipped peer leaders with knowledge, facilitation skills and confidence in conducting group sessions. Several community-based activities were organised outside the peer-group sessions. The inclusion of more group-based activities such as arranging regular physical activity classes for group participants and additional sessions from diabetes experts were recommended as a future improvement.

The assessment of the *participant-level factors* indicates that the program reach was high. The attendance of group sessions was moderate, and the overall program retention rate was quite high. Although the primary outcome in our study did not reach statistical significance, the participants in the intervention arm were more likely to adopt healthy behaviours as compared to their controls. The participants found the overall intervention to be useful in assisting them in adopting healthy lifestyle behaviours, and there was a willingness to continue to maintain these lifestyle behaviours after the program. Inconvenient time and location were reported as the main barriers for participants who did not attend any sessions.

The assessment of the *community-level factors* shows that participants took part in several community activities and had regular contacts with peer leaders and other group members outside group sessions. The communities’ trust in the local partnering institute and the commitment of the local political leaders were important in facilitating the high uptake of the program. Some groups found it challenging to identify suitable community venues for group physical activities.

### Strengths of the K-DPP intervention

Although recruitment through home visits was quite intensive, this did achieve very high participation rates in screening. In our previous experience, recruitment through mailed invitations typically achieves a low response rate [14, 44]. However, in this study, a majority of individuals were willing to join the study. This could be due to the alarmingly high incidence of T2DM in Kerala [6, 7]; hence, the high-risk individuals were keen to participate and viewed the program to be valuable for themselves and their entire families.

The intervention was delivered through 15 sessions as planned. Although in high-income countries settings, intervention intensity as high as 22 sessions is recommended [11, 44]. Tabak et al., in their review of the translation of 44 diabetes prevention programs [21], noted that program implementers often attempted to minimise program delivery costs by employing various strategies, for example, by reducing the number of sessions. Moreover, in resource-constrained settings such as LMICs, it is probably not realistic to use 22 sessions as a benchmark for acceptable program intensity. Furthermore, the relatively short peer leader training was sufficient for successful program implementation when complemented with handbooks for both peer leaders and participants. In our study, the training was perceived effective in equipping lay peer leaders with the knowledge and important skills required to run community-based diabetes prevention group sessions.

Overall, the intervention participants were quite satisfied with the program delivery and with their interactions with peer leaders, who had been able to adopt an autonomy supportive facilitation style, and with other group members. Behaviour change, when seen as a part of the group-based program, has shown to be effective in several studies [44]. In our study, diabetes education through group sessions and community-based activities were proven useful in adopting healthy lifestyle behaviours. Furthermore, despite the high burden of diabetes in the study area, the high-risk individuals did not have access to any specific diabetes prevention resource material in local language prior to the program. Hence, the K-DPP program served as a basis for all educational and informational needs of participants, their families and communities.

The intervention was implemented in strong collaboration with the communities it served. Supported by the local government representatives (Panchayat), trained peer leaders and LRPs, the K-DPP trial utilised strong community linkages. The local partnering institution SCTIMST has a long, trustworthy reputation in the study communities, due to which the program gained its acceptability among local leaders, health professionals and experts. Like previous studies from this institute [45, 46], K-DPP too was well received by participants. Additionally, in some intervention communities, the commitment of the local political leaders emerged as an additional success factor for high uptake of the program.

The participants reported that they shared the knowledge gained through the sessions with other family members. The opportunity for communities to participate in activities organised by the K-DPP team outside the group sessions brought all parties together as a community; hence, non-participant community members benefitted from the intervention as well. At the end of 12 months, many control participants reported that they had made some lifestyle changes.

### The importance of implementation evaluation

Rigorous evaluation should be a central feature of the implementation of such programs. When combined with outcome evaluation, implementation evaluation can contribute to an evidence base for wider implementation and scale-up of research programs, thereby enhancing the potential for population-level impact and for facilitating program translation to other settings and contexts. For example, our recent systematic review on the implementation of diabetes prevention programs demonstrates that lifestyle interventions with only low to moderate frequency (i.e. eight to 14 sessions over the duration of 12 months) but high duration (at least 12 months) can be highly effective in reducing diabetes risk in high-risk individuals, even when weight loss was only ‘low’ or ‘moderate’. This could be very promising especially for resource-constrained settings where large populations need to be reached by such programs [44].

Our findings of the implementation evaluation show that a group-based, peer support program to assist people in reducing their risk of developing diabetes is feasible and acceptable as measured by participants’ satisfaction, perceived support, usefulness and willingness to use the strategies learnt in the future.

In conducting implementation evaluation, while the RE-AIM framework has continued to evolve and has been increasingly used to evaluate and facilitate the translation of research findings, there are some limitations in applying reporting criteria for all five dimensions of RE-AIM [38]. In our view, the RE-AIM definition of individual-level maintenance is equivalent to long-term effectiveness. Traditionally, many research trials do not collect follow-up data beyond 12 months after intervention cessation. Similarly, like other authors, we found the definition of reach and individual-level adoption overlapping, and it is difficult to differentiate between the two [43]. In our view, ‘adoption’ should relate to the willingness to adopt the strategies as part of the intervention (e.g. goal setting, self-monitoring, action planning, etc.).

In a recent systematic review of diabetes prevention programs, we analysed 38 studies, choosing the PIPE Impact Metric for evaluation rather than the RE-AIM [44]. We determined the PIPE framework to be more appropriate, informative and less complex in evaluating the degree of program impact on its objectives. This framework considers both provider-related factors, i.e. penetration and implementation, and participant-related factors, i.e. participation and effectiveness [30]. However, mapping the components from the two models into provider- and participant-level factors, and adding the community-level factors, for this evaluation, allowed us to undertake a comprehensive evaluation of the program implementation.

### Limitations

Our evaluation does have some limitations. Firstly, the behaviour change data reported in this study is self-reported, hence should be used with caution. Secondly, our study did not investigate the factors that may have facilitated healthy lifestyle behaviours in the control communities. Also, several authors have discussed the importance of reporting the implementation fidelity of community-based interventions with some proposing a conceptual framework based on adherence, moderators and intervention agents’ behaviours [22, 26, 47, 48]. In this evaluation, we have focused on a fidelity determined by adherence to protocol only. Further assessment of implementation fidelity is needed to understand all the elements required for a successful implementation. Lastly, some of the data elements reported under community-level factors may seem to be overlapping with the participant-level factors such as participation in community-based activities. However, in order to differentiate between the activities undertaken in group sessions and those conducted outside group sessions (in the community), these are reported separately.

### Implication for practice

The findings of this unique community-based intervention model using low technology and local expertise for reducing diabetes incidence are also relevant and potentially applicable to other LMICs as well as resource-poor settings in high-income countries. The community engagement approach could be highly beneficial to widely implement sustainable lifestyle modifications programs in LMICs. Our findings will be used to inform the future development, adaptation and implementation of diabetes prevention programs to reduce long-term diabetes risk in India and other LMICs. Lessons from this study will also be relevant and have applicability to other rapidly developing low- and middle-income countries with high burdens of type 2 diabetes.

## Conclusion

This comprehensive implementation evaluation from the provider-, participant- and community-level perspective shows that group-based community diabetes prevention programs are feasible and acceptable in changing lifestyle behaviours in high-risk individuals in a LMIC. The community’s trust in the local partnering institute and the commitment of the local political leaders were undoubtedly key success factors. The findings from this evaluation will guide future development, adaptation and implementation of diabetes prevention programs in LMICs.

## Abbreviations

DPES: Diabetes prevention education sessions
IDRS: Indian Diabetes Risk Score
IGT: Impaired glucose tolerance
K-DPP: Kerala Diabetes Prevention Program
LACs: Legislative assembly constituencies
LMICs: Low- and middle-income countries
LRP: Local resource person
OGTT: Oral glucose tolerance test
PIPE: Penetration, implementation, participation, effectiveness
PL: Peer leaders
RCTs: Randomised controlled trials
RE-AIM: Reach, effectiveness, adoption, implementation, maintenance
T2DM: Type 2 diabetes mellitus
